# Immediate and Short-Term Intraocular Pressure Changes Following Intravitreal Injection and Associated Factors

**DOI:** 10.3390/jcm14144821

**Published:** 2025-07-08

**Authors:** Manabu Yamamoto, Kumiko Hirayama, Akika Kyo, Gen Kinari, Yuki Kojima, Takeya Kohno, Shigeru Honda

**Affiliations:** Department of Ophthalmology and Visual Science, Graduate School of Medicine, Osaka Metropolitan University, Osaka 545-8585, Japan; kumiko.hirayama126@hotmail.co.jp (K.H.); mingxiang.ac@gmail.com (A.K.); k56rin@gmail.com (G.K.); yuki.kojima0126@gmail.com (Y.K.); takeyakohno@msn.com (T.K.); shonda@omu.ac.jp (S.H.)

**Keywords:** intravitreal injection, anti-vascular endothelial growth factor, intraocular pressure, ranibizumab, aflibercept, brolucizumab, faricimab

## Abstract

**Objectives**: To evaluate the immediate and short-term changes in intraocular pressure (IOP) following intravitreal injection (IVI) of anti-vascular endothelial growth factor (VEGF) agents and to identify the clinical and procedural factors associated with IOP elevation after treatment. **Methods**: This retrospective study included 118 eyes from 115 patients who underwent IVI with anti-VEGF agents at Osaka Metropolitan University Hospital between September 2024 and January 2025. IOP was measured at three time points, namely before injection, within 1 min after injection, and at 30 min, in selected eyes with a post-injection IOP ≥ 25 mmHg. Differences in IOP elevation were analyzed according to the disease type and anti-VEGF agent. Univariate and multivariate linear regression analyses were performed to identify clinical factors associated with IOP elevation. **Results**: Mean IOP significantly increased from 13.9 ± 3.3 mmHg at baseline to 39.2 ± 12.4 mmHg immediately after injection (*p* < 0.001), with 79.7% of eyes showing an IOP ≥ 25 mmHg. Among those remeasured, IOP decreased to 17.7 ± 6.5 mmHg at 30 min. Significant differences in IOP elevation were observed among anti-VEGF agents (*p* < 0.001), with aflibercept at 2 mg and 8 mg showing greater increases than other agents. Multivariate analysis identified higher baseline IOP, history of glaucoma, absence of prior vitrectomy, and use of aflibercept (2 mg or 8 mg) as significant risk factors for greater post-injection IOP elevation. **Conclusions**: Transient IOP elevation ≥ 25 mmHg was observed in the majority of eyes after IVI but typically resolved within 30 min. Aflibercept use, high baseline IOP, glaucoma history, and absence of prior vitrectomy were associated with greater IOP elevation. Careful monitoring and attention to injection volume may be warranted, particularly in high-risk patients.

## 1. Introduction

Intravitreal injection (IVI) of anti-vascular endothelial growth factor (VEGF) agents has become the standard of care for a variety of retinal diseases, including neovascular age-related macular degeneration (AMD), diabetic macular edema (DME), retinal vein occlusion (RVO), and myopic choroidal neovascularization (mCNV) [1,2,3,4,5,6,7,8,9]. Over the past decade, the frequency of IVI has increased dramatically worldwide, not only due to the growing elderly population but also because of the broadened indications for anti-VEGF therapy. The global demand for anti-VEGF therapy has risen substantially over the past decade, driven in part by the increasing prevalence of age-related retinal diseases in aging populations [10,11]. In Japan, for example, national insurance statistics and real-world clinical data have shown a steady year-on-year increase in IVI procedures, reflecting both the expanding indications for treatment and the growing number of eligible patients. This surge in IVI frequency has also introduced new challenges regarding patient safety, treatment adherence, and healthcare system burden [11].

Although IVI is generally regarded as safe and minimally invasive, it induces a transient increase in intraocular pressure (IOP) immediately after injection due to acute volume expansion within the closed ocular compartment [12,13]. Clinicians are increasingly expected to balance treatment efficacy with procedural safety, and short-term complications like post-injection IOP spikes have garnered renewed attention. Technological advances such as the introduction of prefilled syringes (PFSs) have contributed to procedural efficiency and reduced contamination risks. However, these innovations also bring variability in delivered injection volume, which may unpredictably affect IOP dynamics.

IOP elevation after IVI can be broadly categorized into two types: transient (short-term) and sustained (long-term) [12]. Transient IOP elevation commonly occurs within minutes of injection and generally resolves without intervention within 30 to 60 min [14]. This is mainly due to mechanical volume displacement and is influenced by ocular rigidity and outflow facility [13]. In contrast, sustained elevation is defined as IOP remaining above normal thresholds over multiple follow-up visits, which may persist despite resolution of acute volume changes. The reported incidence of sustained IOP elevation ranges from 3.5% to over 10%, depending on the definition used and the population studied [14,15,16].

While transient IOP spikes are often considered benign in healthy eyes, repeated acute elevations or sustained pressure increases may pose a risk to optic nerve health, especially in eyes with pre-existing glaucoma [17]. Long-term exposure to anti-VEGF therapy has also been associated with chronic trabecular meshwork dysfunction, possibly due to microparticle deposition, protein aggregation, or subclinical inflammation [12,14]. Even transient IOP elevations may impair ocular perfusion and potentially affect optic nerve integrity, particularly in eyes with pre-existing damage.

Several clinical and procedural factors influence the magnitude and duration of post-injection IOP elevation. These include the type and volume of anti-VEGF agent used, the presence or absence of vitreous reflux, syringe design (particularly prefilled syringes), lens status, baseline IOP, glaucoma history, and previous vitrectomy. For instance, aflibercept at 8 mg (0.07 mL) and some prefilled syringe designs have been associated with more pronounced pressure rises [18,19]. Notably, recent innovations in syringe design and drug formulation have further complicated the IOP dynamics following IVI. Prefilled syringes, while advantageous for workflow efficiency and sterility, have been associated with variability in the delivered volume [20]. Moreover, new agents such as faricimab, which target both VEGF-A and angiopoietin-2, may have unique biophysical properties influencing ocular outflow [21]. These nuances necessitate a more comprehensive evaluation of post-injection IOP behavior in real-world settings.

Despite this knowledge, few real-world studies have comprehensively evaluated early post-injection IOP fluctuations across various agents and patient characteristics. Many prior studies have either relied on simulated injection models or included limited time points, often omitting the immediate post-injection phase. The present study therefore aimed to fill this gap by characterizing immediate and short-term IOP changes after IVI in a real-world Japanese clinical setting. It further sought to identify the clinical and procedural factors associated with post-injection IOP elevation. By doing so, we hope to contribute to a more individualized approach to IOP management in patients undergoing anti-VEGF therapy.

## 2. Materials and Methods

### 2.1. Study Design and Ethics

This retrospective observational study was conducted at Osaka Metropolitan University Hospital from September 2024 to January 2025. The study was approved by the Ethics Committee of Osaka Metropolitan University Graduate School of Medicine (Approval No. 2019-062) and adhered to the tenets of the Declaration of Helsinki. Written informed consent was waived due to the retrospective design and anonymization of the data.

### 2.2. Study Participants

A total of 115 patients (118 eyes) who underwent intravitreal injection (IVI) with anti-VEGF agents during the study period were included. Table 1 shows the baseline characteristics of the patients in this study. Inclusion criteria were: (1) a diagnosis of AMD, DME, macular edema (ME) secondary to RVO, mCNV, or neovascular glaucoma (NVG) and (2) the availability of IOP measurements taken before injection, immediately after injection (within 1 min), and at 30 min post-injection in cases where the immediate IOP was ≥25 mmHg. Both treatment-naïve and previously treated eyes were included. Exclusion criteria included patients with recent ocular surgery within the past 3 months, active intraocular inflammation, or a history of uveitis or ocular trauma. Patients with incomplete medical records or inadequate IOP documentation were also excluded from the analysis.

### 2.3. IVI Procedure

The following intravitreal anti-VEGF medications were used: ranibizumab (Lucentis^®^, Novartis Pharma AG, Basel, Switzerland; Genentech, Inc., South San Francisco, CA, USA) and ranibizumab biosimilar (Ranibizumab BS^®^, Senju Pharmaceutical Co., Ltd., Kobe, Japan) (IVR), aflibercept at 2 mg and 8 mg (Eylea^®^, Bayer AG, Leverkusen, Germany) (IVA2, IVA8), brolucizumab (Beovu^®^, Novartis Pharma AG, Basel, Switzerland) (IVBR), and faricimab (Vabysmo^®^, Hoffmann-La Roche Ltd., Basel, Switzerland; Genentech, Inc., South San Francisco, CA, USA) (IVF). All injections were performed under topical anesthesia using a 30-gauge needle. The injection site was located 3.5 mm posterior to the corneal limbus in either the superotemporal or inferotemporal quadrant. No anterior chamber paracentesis was performed before or after injection. The injection volume was 0.05 mL for all agents, except for IVA8, which was administered at 0.07 mL. PFSs were used for all agents except IVA8 and IVF. The procedure followed standard intravitreal injection techniques as described previously. To control for technique variability, all injections were administered by IVI specialists using a consistent injection protocol, including identical needle entry angle, injection speed, and post-injection ocular massage avoidance. Eyes were examined under slit-lamp biomicroscopy immediately post-injection to assess for complications.

### 2.4. IOP Measurement

IOP was measured using a non-contact tonometer (NCT) (NT-530P, NIDEK Co., Ltd., Gamagori, Japan). Each eye was measured three times, and either the mode (most frequent value) or the median of the three readings was used for analysis. IOP was recorded at three time points, namely before intravitreal injection (baseline), within 1 min after injection (post-injection), and 30 min after injection (30 min), in eyes with a post-treatment IOP of ≥ 25 mmHg.

### 2.5. Outcome Measures

The proportion of eyes with an IOP exceeding 25 mmHg at each time point was assessed, and IOP changes 30 min after injection were analyzed. Differences in post-injection IOP elevation and the proportion of eyes exceeding 25 mmHg were also evaluated according to disease type and anti-VEGF agent. To investigate factors associated with IOP elevation, the following variables were evaluated: age; sex; history of hypertension, diabetes mellitus, and smoking; history of cataract surgery, vitrectomy, and glaucoma; number of previous IVA injections; baseline IOP; type of underlying retinal disease; and type of anti-VEGF agent used. Although a small number of patients received bilateral injections, the procedures were not performed simultaneously. Moreover, the contralateral eyes often had differing underlying conditions or treatment histories, making them unsuitable for use as internal controls. Therefore, we used each eye’s baseline IOP as the reference for evaluating post-injection changes.

### 2.6. Statistical Analysis

Repeated measures analysis of variance (ANOVA) was used to evaluate IOP changes across three time points—baseline, immediately post-injection, and 30 min post-injection. One-way ANOVA was used to compare the amount of post-injection IOP elevation (i.e., the change from baseline) across drug groups. Chi-square tests were used to assess categorical differences in the proportion of eyes with IOP ≥ 25 mmHg. Chi-square tests were applied with caution, as subgroup sizes in some comparisons were limited. In cases where expected frequencies in cells were <5, interpretations were made conservatively, and these limitations are discussed accordingly. For multiple comparisons in the univariate analysis, a false discovery rate (FDR) correction using the Benjamini–Hochberg method was applied. Univariate and multivariate linear regression analyses were conducted to identify factors associated with post-injection IOP elevation, including age, sex, disease type, baseline IOP, history of glaucoma or vitrectomy, lens status, and drug type. For the multivariate analysis, variables with the lowest *p* values in the univariate analysis were selected according to the number of cases divided by 15, thereby limiting the number of included covariates to avoid model overfitting. Statistical analyses were conducted using SPSS version 24.0 (IBM Japan, Ltd., Tokyo, Japan), in which *p* values < 0.05 were regarded as significant. A summary of the dataset used for statistical analysis is provided in the Supplementary Materials (Table S1).

## 3. Results

**Figure 1:**
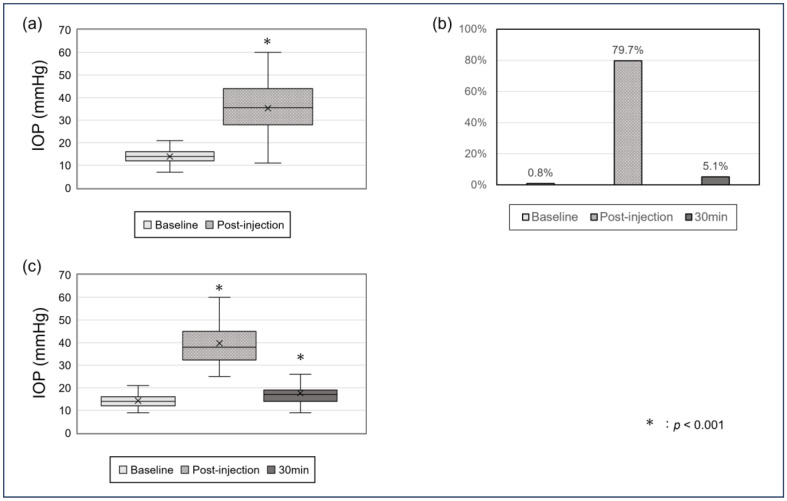
Immediate and short-term intraocular pressure (IOP) changes following intravitreal injection (IVI). (**a**) Boxplots showing IOP values at baseline and immediately after intravitreal injection in all treated eyes (n = 118). (**b**) Bar chart showing the proportion of eyes with IOP ≥ 25 mmHg at each time point (n = 94). (**c**) Boxplots showing IOP values at baseline, immediately post-injection, and at 30 min post-injection in eyes with initial an IOP ≥ 25 mmHg (n = 94).

The mean IOP increased significantly from 13.9 ± 3.3 mmHg at baseline to 39.2 ± 12.4 mmHg at the post-injection time point (*p* < 0.001) (Figure 1a). At this time point, 94 eyes (79.7%) had an IOP of ≥25 mmHg, which decreased markedly to 6 eyes (5.1%) at 30 min among those remeasured (Figure 1b). To illustrate IOP trends in this high-risk subgroup, Figure 1c presents the IOP changes in eyes with an immediate post-injection IOP ≥ 25 mmHg. In these eyes, the mean IOP was 14.3 ± 3.3 mmHg at baseline, increased to 39.7 ± 9.6 mmHg immediately after injection, and then decreased to 17.7 ± 6.5 mmHg at 30 min. A repeated measures ANOVA demonstrated significant differences among the three time points (*p* < 0.001), with all pairwise comparisons also showing significant differences (baseline vs. post-injection, baseline vs. 30 min, and post-injection vs. 30 min; all *p* < 0.001). IOP at 30 min remained significantly higher than at baseline (*p* < 0.001).

**Figure 2:**
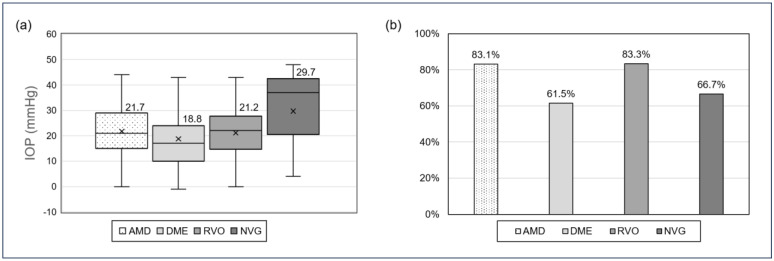
Post-injection IOP elevation and proportion of eyes with IOP ≥ 25 mmHg stratified by underlying retinal disease. (**a**) Boxplots showing the distribution of IOP elevation (post-injection minus baseline) across four disease groups. (**b**) Bar chart showing the proportion of eyes with post-injection IOP ≥ 25 mmHg for each disease group.

Regarding the amount of post-injection IOP elevation, no significant differences were observed among disease types (excluding mCNV, which included only one eye; *p* = 0.505) (Figure 2a). Similarly, the proportion of eyes with IOP ≥ 25 mmHg did not differ significantly among these disease groups (*p* = 0.287) (Figure 2b).

**Figure 3:**
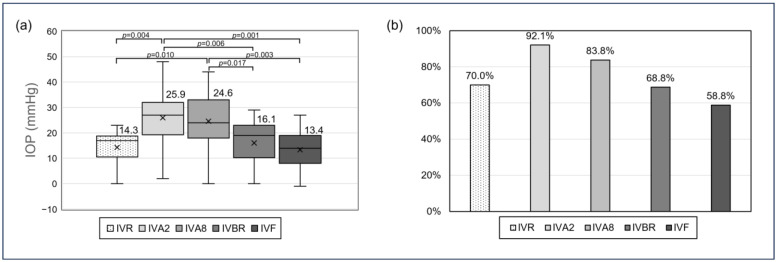
Post-injection IOP elevation and proportion of eyes with IOP ≥ 25 mmHg stratified by anti-VEGF agent. (**a**) Boxplots showing the distribution of IOP elevation (post-injection minus baseline) across five anti-VEGF agents. (**b**) Bar chart showing the proportion of eyes with IOP ≥ 25 mmHg at the post-injection time point for each anti-VEGF agent.

One-way ANOVA revealed a statistically significant difference in post-injection IOP elevation among the five anti-VEGF agents (*p* < 0.001) (Figure 3a). Post hoc pairwise comparisons with FDR adjustment showed that aflibercept at both 2 mg (IVA2) and 8 mg (IVA8) was associated with significantly greater IOP elevations compared with ranibizumab (IVR; *p* = 0.004 and 0.010, respectively), brolucizumab (IVBR; *p* = 0.006 and 0.017), and faricimab (IVF; *p* = 0.001 and 0.003). No significant differences were observed among IVR, IVBR, and IVF. IVA2 and IVA8 showed comparable IOP elevations (*p* = 0.601). While the overall proportion of eyes with IOP ≥ 25 mmHg also differed significantly among the five drug groups (*p* = 0.035), no significant differences were detected in pairwise comparisons after correction for multiple testing (Figure 3b).

Univariate and multivariate regression analyses identified several factors significantly associated with greater IOP elevation at the post-injection time point. These included a history of vitrectomy (coefficient (Co) = −11.331; 95% confidence interval (CI), −16.450, −6.212; *p* < 0.001), history of glaucoma treatment (Co = 8.731; 95% CI, 2.266, 15.196; *p* = 0.009), baseline IOP (Co = 0.887; 95% CI, 0.346, 1.428; *p* = 0.002), and use of IVA2 (Co = 10.383; 95% CI, 3.856, 16.910; *p* = 0.002) and IVA8 (Co = 12.007; 95% CI, 5.452, 18.562; *p* < 0.001). The final multivariate model explained 40.2% of the variance in IOP elevation (adjusted R^2^ = 0.402, *p* < 0.001). The variance inflation factor (VIF) values were all < 2.0, indicating low collinearity among the selected covariates. No significant interactions were detected among the predictors (Table 2).

## 4. Discussion

Our study revealed that IOP increased significantly immediately after intravitreal injection (IVI) of anti-VEGF agents, with nearly 80% of eyes exhibiting an IOP of ≥25 mmHg. However, this rise was transient, and most eyes returned to near-baseline levels within 30 min. These findings are consistent with previous reports indicating that short-term IOP spikes following IVI are common but usually self-limiting and do not require intervention [12,19,21,22]. Since IOP was remeasured at 30 min only in eyes with post-injection IOP ≥ 25 mmHg, our dataset is subject to selection bias, and the analysis of recovery trends is limited to this subset of patients. As a result, the true rate of IOP normalization in the overall population may have been underestimated. This limitation should be addressed in future studies with standardized follow-up across all cases, regardless of the initial IOP. Nonetheless, the mean post-injection IOP in our study (39.2 mmHg) was substantial and could affect eyes with fragile optic nerves, particularly those with a history of glaucoma [17]. We also acknowledge that IOP was measured only at three time points: baseline, immediately after injection, and, in selected cases, at 30 min post-injection. The 30 min time point was chosen on the basis of clinical experience suggesting that IOP typically returns to near-baseline levels by that time [14]. While intermediate time points such as 10 or 20 min could offer more detailed temporal insights, routine measurement at such intervals is often impractical in real-world clinical settings. Future prospective studies incorporating more frequent IOP measurements may help further characterize the dynamic changes in post-injection IOP.

When stratified by disease type, we found no statistically significant differences in IOP elevation. However, eyes with NVG tended to show greater IOP changes. As discussed later, a history of glaucoma was also identified as a significant risk factor for post-injection IOP elevation in our multivariate analysis. This may be attributable to compromised outflow facility and structural damage to the trabecular meshwork, which can limit the eye’s ability to compensate for acute volume increases following intravitreal injection [17]. MCNV was not included in this comparison, as only one case was present in the cohort. Increased scleral compliance in highly myopic eyes may contribute to attenuated IOP responses, consistent with prior reports on ocular rigidity [23,24]. Further studies with larger cohorts are needed to clarify disease-specific responses.

Notably, we observed significant differences in post-injection IOP among anti-VEGF agents. Both aflibercept at 2 mg (IVA2) and aflibercept at 8 mg (IVA8) were associated with a higher proportion of eyes showing IOP ≥25 mmHg compared with other agents—92.1% and 83.8%, respectively. While the larger injection volume of IVA8 (0.07 mL vs. 0.05 mL) likely contributed to the IOP increase, it is noteworthy that IVA2, despite having a smaller nominal volume, resulted in a slightly higher IOP elevation rate. One possible explanation is the use of PFSs for IVA2. Prior studies, including Gallagher et al., have shown that aflibercept PFSs may deliver more than the intended 0.05 mL due to design variability, with 21% of injections exceeding 0.07 mL [18,20]. This unintended over-delivery could result in higher IOP spikes. In contrast, IVA8 may benefit from more consistent volume control despite its higher labeled dose. These findings support the notion that injected volume—and its variability—is a key determinant of post-injection IOP elevation [13]. Our regression analysis further demonstrated that higher baseline IOP, a history of glaucoma, and absence of prior vitrectomy were significant independent predictors of greater IOP elevation. These findings agree with previous literature suggesting that eyes with impaired aqueous outflow or intact vitreous bodies are less capable of rapidly compensating for acute volume changes [17,25]. It should be noted that the sample sizes for some anti-VEGF agents, such as ranibizumab, brolucizumab, and faricimab, were relatively small compared with aflibercept. Although FDR correction was applied, these imbalances may limit the statistical power of inter-group comparisons. Larger prospective studies are warranted to validate the observed trends.

In addition to the use of IVA2 and IVA8, our multivariate analysis identified several clinical risk factors for higher post-injection IOP, including higher baseline IOP, a history of glaucoma, and absence of prior vitrectomy. Notably, baseline IOP and a history of glaucoma may reflect a similar underlying pathology—namely, impaired aqueous outflow. Eyes with reduced outflow facility may be less able to compensate for sudden intraocular volume changes, thereby experiencing greater IOP spikes following injection [17]. Conversely, eyes with prior vitrectomy likely have lower vitreous resistance, which may facilitate posterior fluid displacement and reduce the magnitude of anterior chamber pressure elevation [25]. In our dataset, pseudophakia was not significantly associated with IOP elevation; however, previous studies have suggested that pseudophakic eyes may exhibit smaller IOP increases, possibly due to altered anterior segment dynamics [26]. The number of prior injections did not significantly influence post-injection IOP changes, suggesting that cumulative exposure may not be a major determinant of acute IOP response in the short term. Vitreous reflux, though not formally measured in this study, is known to reduce IOP spikes by allowing partial fluid escape immediately after injection [27,28]. Prospective evaluations of reflux, as well as the role of pseudophakia and other anatomical factors, may offer further insight into modifiable contributors to post-injection IOP elevation.

Corneal biomechanical properties such as corneal hysteresis (CH) and central corneal thickness have been implicated in modulating IOP responses to mechanical stimuli. Eyes with low CH or thinner corneas may exhibit greater IOP spikes due to diminished shock-absorbing capacity. Although these parameters were not evaluated in our study, future research incorporating such metrics may elucidate their contribution to post-injection IOP dynamics, especially in glaucoma-prone populations [24,29]. The clinical significance of injection volume variability is underscored by case reports of severe complications such as transient central retinal artery occlusion linked to over-delivery from PFSs [20]. This reinforces the necessity of quality control in syringe manufacturing and highlights the need for standardization across institutions. Employing precise dosing techniques, including dead space calibration and volume verification, may mitigate the risk of adverse pressure-related outcomes.

These insights have practical implications for enhancing procedural safety in real-world clinical practice. In patients with known risk factors such as glaucoma or high baseline IOP, individualized strategies—such as optimizing topical IOP-lowering medications prior to injection or considering prophylactic anterior chamber paracentesis—may help minimize acute pressure spikes. Furthermore, increased awareness of injection volume variability, especially in prefilled syringes, should prompt clinicians to adopt standardized injection techniques and consider volume verification protocols where feasible. The findings of this study also underscore the potential utility of incorporating immediate post-injection IOP measurement into routine practice, particularly in high-risk patients. Real-time IOP assessment may facilitate timely intervention and help prevent rare but serious complications. Looking ahead, the development of predictive models and automated monitoring systems that incorporate biometric and procedural variables could support more personalized and safer IVI protocols. Given the growing volume of intravitreal procedures worldwide, ensuring consistent safety standards will be essential for sustainable long-term treatment of retinal diseases. Moreover, raising awareness among clinicians and improving procedural education may further enhance injections’ safety.

Our study has several limitations. Due to its retrospective nature, selection bias and variability in the injection technique cannot be ruled out. IOP at 30 min was only measured in eyes with IOP ≥ 25 mmHg immediately after injection, which precluded comprehensive analysis of IOP changes at that time point across the full study population. Another limitation of this study is the use of an NCT for IOP measurement, which may introduce variability compared with Goldmann applanation tonometry (GAT), the clinical gold standard. However, to minimize measurement error and enhance reliability, we performed three consecutive IOP readings for each eye and used the average value for analysis. Future studies incorporating cross-validation with GAT or other tonometry methods would help ensure further accuracy and comparability. Additionary, the sample size for some subgroups, particularly NVG and mCNV, was relatively small, which may limit the statistical power. Due to the small sample sizes in certain subgroups such as NVG and mCNV, the statistical power to detect differences was limited, and the generalizability of findings for these groups should be interpreted with caution. Lastly, biometric data such as axial length, anterior chamber depth, vitreous reflux and CH, which may influence IOP dynamics, were not collected [27,28,29]. These ocular structural parameters could potentially affect the IOP response following injection and warrant further investigation. Furthermore, the study did not include OCT-based assessment of the optic nerve, as our primary focus was on short-term IOP changes. However, evaluating whether transient IOP spikes affect the optic nerve’s morphology would be clinically valuable. Future studies should consider incorporating both longitudinal OCT measurements and biometric parameters such as CCT, CH, and axial length to more comprehensively elucidate the anatomical and biomechanical factors influencing post-injection IOP dynamics. Despite these limitations, our findings provide valuable real-world insights into short-term IOP fluctuations after IVI in a Japanese population, highlighting the influence of drug type, ocular history, and anatomical factors. Clinicians should be aware of these risk factors and consider them in post-injection monitoring protocols, particularly in patients with glaucoma or a high baseline IOP.

## 5. Conclusions

Post-injection IOP elevation ≥ 25 mmHg occurred in 79.7% of eyes, with the highest rates in those treated with aflibercept at 2 mg (92.1%) and 8 mg (83.8%). However, IOP typically returned to near-baseline levels within 30 min. The use of aflibercept (especially via prefilled syringes), a higher baseline IOP, a history of glaucoma, and absence of prior vitrectomy were significantly associated with greater IOP elevation. These findings emphasize the importance of identifying high-risk patients and tailoring post-injection management accordingly. Particular attention should be given to the injection volume’s accuracy, choice of syringe device, and consideration of ocular surgical history. For patients with known glaucoma or elevated baseline IOP, short-term monitoring and preventive strategies—such as paracentesis or volume adjustment—may help mitigate IOP spikes. Future prospective studies incorporating biometric parameters and direct comparisons of injection systems may further refine our understanding of injection-related IOP dynamics. Until then, careful procedural standardization and risk stratification remain key to minimizing IOP-related complications in clinical practice.

## Figures and Tables

**Table 1:** Patient characteristics.

Characteristics	
Number of eyes, eyes	115 of 118 cases
Age, years	75.2 ± 10.3
Gender (male), eyes	75 (63.6)
Hypertension, eyes	64 (54.2)
Diabetes mellitus, eyes	37 (31.4)
Smoking, eyes	60 (50.8)
History of cataract surgery, eyes	65 (55.1)
History of vitrectomy, eyes	15 (12.7)
Number of IVI, times	26.4 ± 25.2
Type of disease, eyes	
AMD	83 (70.3)
mCNV	1 (0.8)
DME	13 (11.0)
ME secondary to RVO	18 (15.3)
NVG	3 (2.5)
Type of medications, eyes	
Ranibizumab (IVR)	10 (8.5)
Aflibercept 2 mg (IVA)	38 (32.2)
Aflibercept 8 mg (IVA8)	37 (31.4)
Brolucizumab (IVBR)	16 (13.6)
Faricimab (IVF)	17 (14.4)

**Table 2:** Univariate and multivariate analysis of factors associated with the amount of post-injection IOP.

Characteristics	Univariate			Multivariate		
	Co	95% CI	*p* Value	Co	95% CI	*p* Value
Age: years	−0.900	(−5.217–3.417)	0.680			
Gender: male	−0.071	(−0.274–0.132)	0.491			
Hypertension	0.771	(−3.400–4.943)	0.715			
Diabetes mellitus	−3.036	(−7.483–1.411)	0.179	−2.418	(−6.349–1.512)	0.225
Smoking	−2.473	(−6.472–1.526)	0.223			
History of cataract surgery	−2.699	(−6.849–1.452)	0.200			
History of vitrectomy	−10.289	(−16.238–−4.341)	0.001	−11.331	(−16.450–−6.212)	0.000
History of glaucoma treatment	9.635	(2.382–16.888)	0.010	8.731	(2.266–15.196)	0.009
Number of IVIs	−0.024	(−0.107–0.059)	0.569			
Baseline IOP	0.678	(0.051–1.304)	0.034	0.887	(0.346–1.428)	0.002
Type of disease						
AMD	0.709	(−3.805–5.223)	0.756			
DME	−2.936	(−9.555–3.683)	0.382			
ME secondary to RVO	−0.253	(−6.036–5.530)	0.931			
NVG	8.501	(−4.615–21.618)	0.202			
Type of medications						
Ranibizumab	−7.737	(−15.066–−0.408)	0.039	-		-
Aflibercept 2 mg	6.735	(2.461–11.009)	0.002	10.383	(3.856–16.910)	0.002
Aflibercept 8 mg	4.681	(0.283–9.079)	0.037	12.007	(5.452–18.562)	0.000
Brolucizumab	−6.153	(−12.120–−0.186)	0.043	2.431	(−4.904–9.767)	0.513
Faricimab	−9.380	(−15.044–−3.715)	0.001	1.428	(−5.841–8.698)	0.698

## Data Availability

The data presented in this study are available in the Supplementary Materials.

## Supplementary Materials

The following supporting information can be downloaded at https://www.mdpi.com/article/10.3390/jcm14144821/s1.
